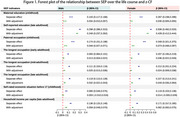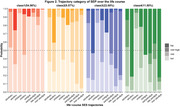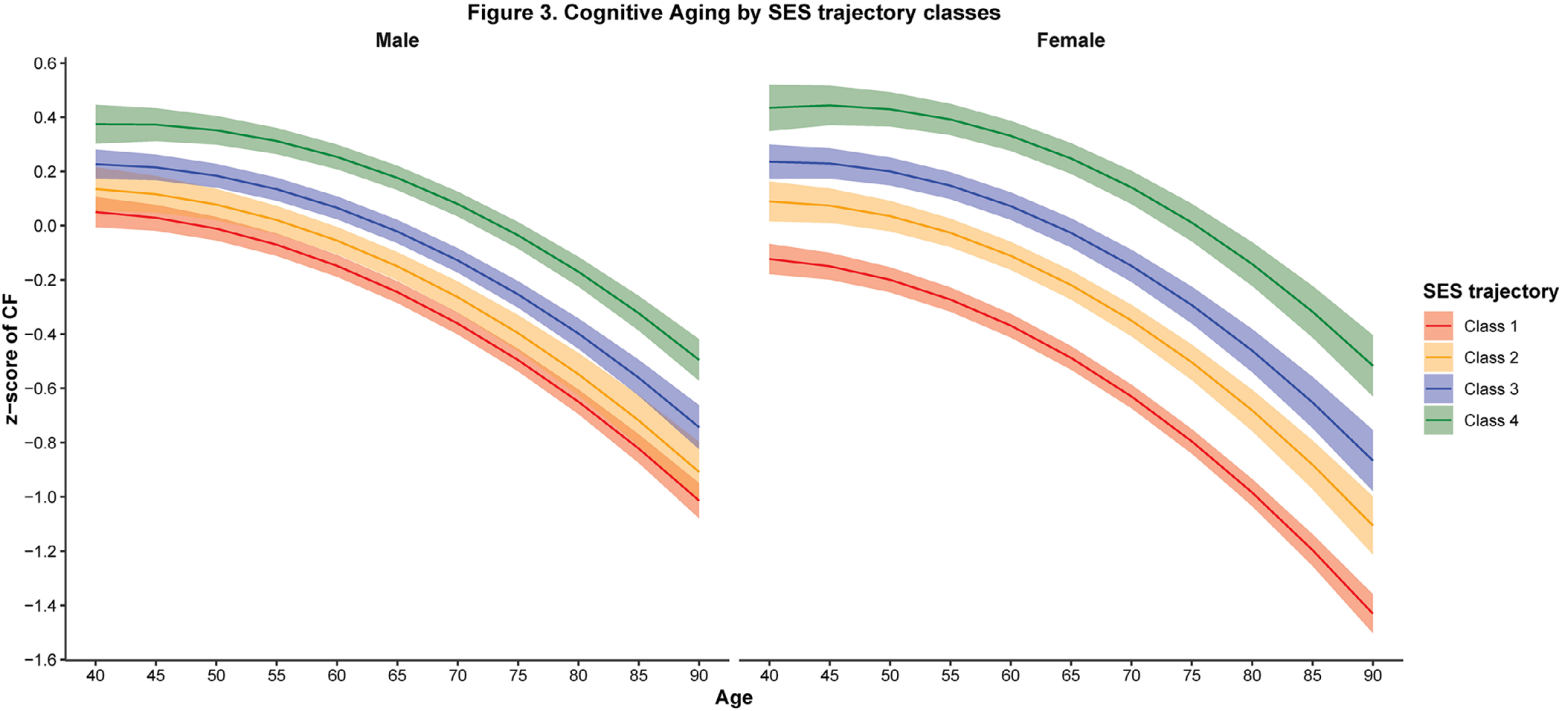# The Impact of Life‐Course Socioeconomic Trajectories on Late‐Life Cognitive Aging among Chinese people

**DOI:** 10.1002/alz70860_101904

**Published:** 2025-12-23

**Authors:** Mingyue Gao, Wenxin Cai, Ya Fang

**Affiliations:** ^1^ Health and Aging Research Center, School of Public Health, Xiamen University, Xiamen, Fujian, China; ^2^ Center for Aging and Health Research, School of Public Health, Xiamen University, Xiamen, Fujian, China; ^3^ Health and Aging Research Center, School of Public Health, Xiamen University, Xiamen, China, Xiamen, Fujian, China; ^4^ National Institute for Data Science in Health and Medicine, Xiamen University, Xiamen, Fujian, China

## Abstract

**Background:**

Socioeconomic status (SES) is a recognized protective factor for cognitive function (CF). However, the critical life stage for SES's impact on CF remains debated and may differ across various SES indicators. Given the rapid societal development in China, this study aims to delineate life‐course SES trajectories and investigate their longitudinal effects on cognitive aging among Chinese people.

**Method:**

The study sample comprised participants aged 40 and older (*N* = 20,055) with longitudinal follow‐ups from the China Health and Retirement Longitudinal Study between 2011 and 2020. SES was defined as education, occupation, and income from childhood to late adulthood. The primary outcome was the z‐score of a global cognitive composite score.

Sankey diagrams were used to illustrate the shifts in life‐course SES mobility over decades. Mixed‐effects models were then conducted to explore the relationship between SES indicators across life stages and late‐life cognitive decline. Latent class analysis was utilized to identify distinct life‐course SES trajectories, with subsequent descriptions of the cognitive decline for each SES trajectory by gender.

**Result:**

Overall, educational attainment exhibited substantial intergenerational upward mobility, followed by moderate upward income mobility, while occupational patterns remained relatively stable in recent decades. All SES indicators were positively correlated with late‐life CF after controlling for covariates, with adulthood education, paternal occupation, and adulthood income demonstrating greater protective effects than SES measured at other life stages. Compared to the consistently low‐level trajectory, individuals from stable high or upward SES trajectory groups experienced better late‐life CF and slower rates of cognitive decline, with these effects being more significant in females than males.

**Conclusion:**

Over the past decades, older Chinese adults have experienced upward trends in education and income mobility, with relatively stable occupational changes. Life‐course education, paternal occupation, and adulthood income are more strongly associated with CF than other life‐stage SES measures. Furthermore, stable high and upward SES trajectories protect against cognitive decline, particularly among women. This study emphasizes that enhancements in lifelong education, occupation, and income may help mitigate the risk of cognitive aging in later life, especially for Chinese women.